# Cough rhythms in asthma: Potential implication for management

**DOI:** 10.1016/j.jaip.2018.12.020

**Published:** 2019

**Authors:** Sirat Lodhi, Jaclyn A. Smith, Imran Satia, Kimberley J. Holt, Robert J. Maidstone, Hannah J. Durrington

**Affiliations:** a University of Manchester Medical School, Manchester, United Kingdom; b Division of Infection, Immunity, Respiratory Medicine, School of Biological Sciences, Faculty of Biology, Medicine, Health, Manchester Academic Health Science Centre, The University of Manchester, Manchester, United Kingdom; c Wythenshawe Hospital, University of Manchester NHS Foundation Trust, Manchester, United Kingdom; d Division of Respirology, McMaster University, Hamilton, Ontario, Canada; e Division of Diabetes, Endocrinology and Gastroenterology, School of Medical Sciences, Faculty of Biology, Medicine, Health, The University of Manchester, Manchester, United Kingdom

To The Editor:

**Clinical Implications**

• Cough frequency in asthma is actually reduced during the night, as in healthy individuals, and significantly increased at the time of waking. Commonly used asthma symptom questionnaires should reflect this. Cough-suppressant medication should be active on waking.

Increasing nocturnal cough signals worsening asthma and treatment escalation. Diurnal variation in airway resistance peaking at around 4:00 am may cause increased early morning symptoms. However, objective cough frequency measurements suggest that mean nighttime cough frequency is actually lower than mean cough frequency during the daytime. Hourly cough frequency would provide more information about the fluctuation in cough rates over 24 hours and could provide an insight into the mechanisms controlling cough in asthma. We analyzed hourly cough frequency data in adults with mild/moderate asthma recorded over 24 hours by the VitaloJAK cough monitor (Vitalograph Ltd, Buckingham, United Kingdom) and compared with that in healthy controls.

Patients with asthma cough significantly more than do healthy individuals over 24 hours, and least between 2:00 and 5:59 am (antiphase to airway resistance). Patients with asthma with nocturnal cough demonstrate greatest increases in cough on waking, increased use and higher doses of inhaled corticosteroids (ICSs), and poorer asthma control. The central suppressive effect of sleep on cough may delay cough presentation until waking.

Nighttime symptoms are common in asthma,^1^ triggering escalation of treatment. Nocturnal symptoms (wheeze, breathlessness, and cough) may be caused by increased airway narrowing and eosinophilic airway inflammation seen in individuals with asthma at 4 am.^2^, ^3^ However, recent objective cough frequency measurements suggest that cough in asthma is reduced at nighttime.^4^ Furthermore, cough threshold might vary by time of day^5^ and cough caused by angiotensin-converting enzyme inhibitors can be reduced by taking the dose in the evening rather than the morning.^6^ It is crucial to understand how cough varies by time of day to gain a better understanding of cough mechanisms. Treatments for cough may be more efficacious if given at the right time of day (chronotherapy). We analyzed hourly cough frequency data recorded in adults with mild/moderate asthma over 24 hours and compared it with that in healthy controls.

Cough recording data from patients with mild/moderate asthma and healthy participants collected during a previous study^7^ were analyzed. Briefly, subjects with a physician's diagnosis of asthma were recruited but not selected for symptoms of cough. Treatment with salbutamol as required and/or ICSs at 500 μg or less of fluticasone propionate equivalent daily with or without a long-acting bronchodilator was permitted. Subjects with uncontrolled symptoms according to the Global Initiative for Asthma classification or not receiving stable medication over the previous 4 weeks were excluded. Healthy control subjects approximately matched for age were also recruited. We excluded current smokers, those with a recent chest infection or exacerbation, and those using any medication that might alter the cough responses (eg, opiates, gabapentin, anticholinergics, and theophylline).^7^ Patients' demographic data and 24-hour cough frequency data from VitaloJAK cough monitor were analyzed using GraphPad prism (version 7.00, GraphPad Software, La Jolla, Calif) and SPSS software (version 25.0, IBM Corporation, Armonk, NY). Mann-Whitney *U* and chi-square tests were used. Median and interquartile ranges are reported. Frequency bar graphs of mean ± SEM rather than median were used to display 4-hour cough frequency data due to multiple values of 0 skewing the data. Subgroup analysis of the asthma group compared individuals with and without nocturnal cough. Nocturnal cough was identified using audio recording and defined as at least 1 cough between retiring to bed and waking. Multivariate logistic regression models were used to identify potential differences in patient characteristics between asthma groups. Spearman rank correlation and the chi-square and Kruskal-Wallis tests were performed.

Data from 92 individuals with asthma and 44 healthy participants were analyzed. Five individuals with asthma and 3 healthy participants were omitted because of incomplete 24-hour cough recordings. Asthma and healthy groups were well matched for sex, body mass index, and smoking pack-years. Participants with asthma were significantly younger (23 years [21-27] compared with 38 years [29-51]) and had a lower percent predicted FEV_1_ (Table I).

**Table I:** Asthma and healthy group patient demographics

Characteristic	Asthma (n = 92)	Healthy (n = 44)	*P* value
Age (y)	23.00 (21.00-27.00)	38.00 (29.00-50.75)	<.0001
Sex (male:female)	39:53	14:30	.26
BMI (kg/m^2^)	24.15 (21.80-27.05)	24.60 (22.30-28.28)	.34
FEV_1_ (% predicted)	95.50 (87.00-103.00)	103.00 (96.25-114.00)	<.0001
FVC (% predicted)	103.00 (95.00-109.00)	104.50 (98.25-118.00)	.05
Age of onset (y)	7.50 (4.25-14.75)		
Serum eosinophils (× 10^9^/L)	0.21 (0.13-0.32)		
Serum total IgE (kU/L)	200.00 (61.50-487.50)		
Feno (ppb)	36.00 (22.00-86.75)		
Atopic, %	80.43		
Methacholine PC_20_ (mg/mL)	0.97 (0.26-3.21)		
SABA use, %	100.00		
ICS use, %	34.78		
ICS/LABA combination use, %	17.39		
Daily ICS dose (μg of FP equivalent)	50.00 (0.00-237.50)		
Steroid naive, %	48.91		
ACQ7 category, %			
Well controlled	53.26		
Partly controlled	35.87		
Poorly controlled	10.87		
GINA category, %			
Well controlled	53.26		
Partly controlled	46.74		

*ACQ7*, 7-Item Asthma Control Questionnaire; *BMI*, body mass index; *FP*, fluticasone proprionate; *FVC*, forced vital capacity; Feno, fraction of exhaled nitric oxide; *GINA*, Global Initiative for Asthma; *LABA*, long-acting beta-agonist; *SABA*, short-acting beta-agonist.

Data quoted as median and interquartile ranges (significance: *P* < .05).

**Figure 1:**
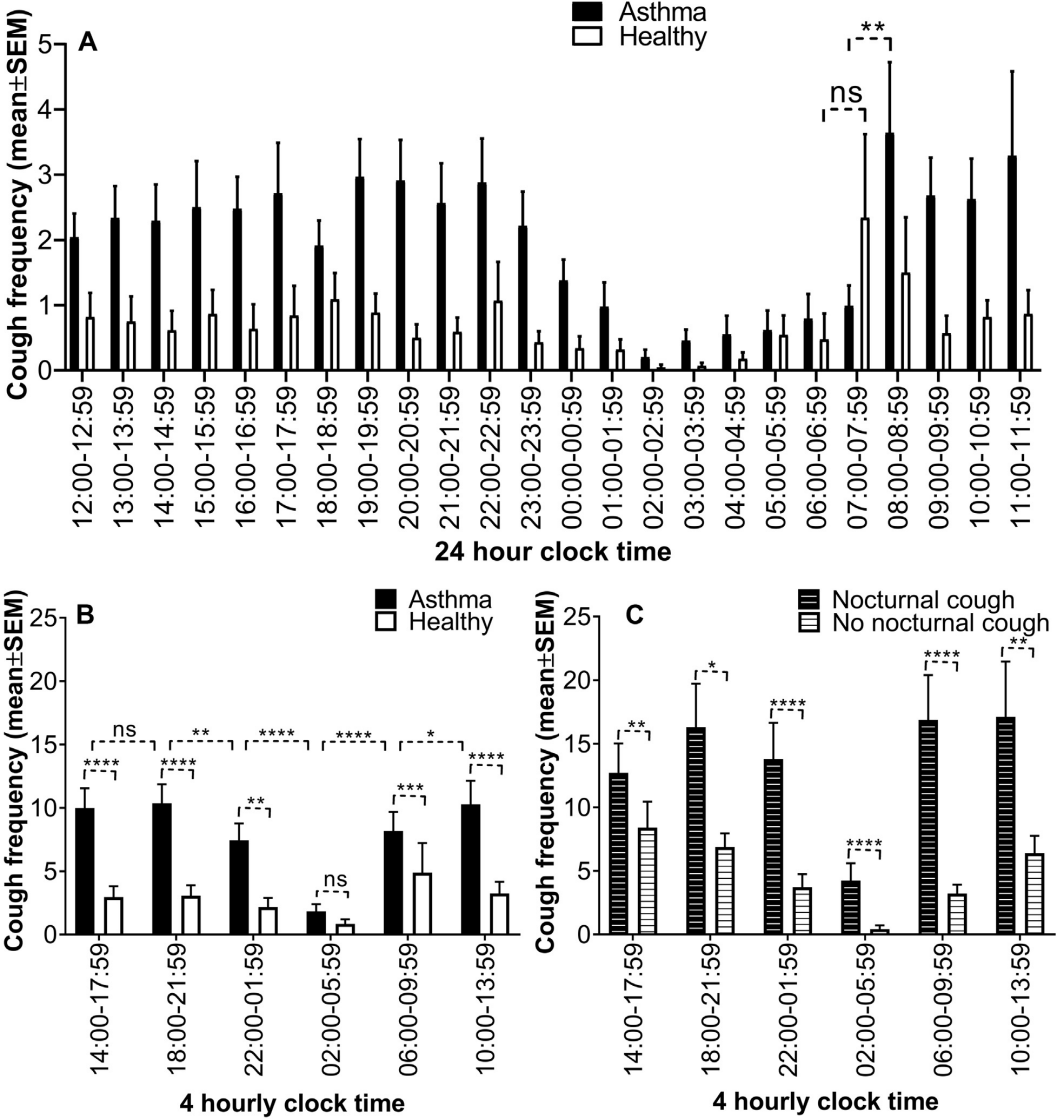
Twenty-four-hour cough frequency (± SEM) for asthma and healthy groups. **A**, Hourly mean cough frequency in asthma and healthy groups. **B**, Four-hourly mean cough frequency in asthma and healthy groups. **C**, Asthma subgroups of individuals with and without nocturnal cough. *ns*, Not significant.

Participants with asthma had significantly greater cough frequency than healthy participants over 24 hours (27 [12-67] vs 3.5 [1-19.75] coughs/24 h respectively; *P* < .0001). Analysis of hourly cough frequency data revealed a similar shaped graph for both healthy individuals and patients with asthma, with significantly more coughs occurring during the day than at night in both groups (*P* < .0006 healthy; *P* < .0001 asthma). There was no discernible rhythm in cough frequency during the daytime in either group. The cough frequency reduced between 00:00 and 00:59 am, reaching a nadir between 02:00 and 02:59 am in the asthma group. There was a significant increase in morning cough frequency in the asthma group, between 08:00 and 08:59 am (*P* = .003), but not in the healthy group (Figure 1, *A*). The data were also analyzed in 4-hour blocks; the asthma group demonstrated significantly greater cough frequency than did the healthy group in all 4-hour blocks except during 2:00-5:59 am (Figure 1, *B*). For both groups, the lowest mean cough frequency was observed in this 2:00-5:59 am block (encompassing the 4:00 am time-point). In the asthma group, this block had a significantly lower mean cough frequency (*P* < .0001) compared with all other blocks. Interestingly, decrease in cough frequency was seen at the expected onset of sleep (22:00 pm to 01:59 am; *P* = .002) in the asthma group, and a further decrease to a nadir at 02:00 to 05:59 am (*P* < .0001). Then followed an increase in cough frequency at the expected time of waking (06:00-09:59 am) (*P* < .0001; Figure 1, *B*). Subgroup analysis of the asthma group demonstrated 34 individuals with nocturnal cough and 58 without. Both groups were well matched for age, sex, body mass index, spirometry, atopy, and 7-item Asthma Control Questionnaire/Global Initiative for Asthma category. Individuals with asthma with nocturnal cough coughed significantly more than did those without nocturnal cough in all 4-hour blocks, with the lowest cough frequency being during the 2:00-5:59 am block centered on 4:00 am in both groups (Figure 1, *C*).

Those with nocturnal cough were more likely to use an ICS (*P* = .02) and to use higher daily doses of ICS (*P* = .02). Univariate logistic regression analysis found that nocturnal cough was associated with higher odds of ICS use (odds ratio [OR], 2.87; 95% CI, 1.17-7.00; *P* = .021) and daily ICS dose (OR, 1.00; 95% CI, 1.00-1.01; *P* = .042). Multivariate logistic regression analysis confirmed the significance of daily ICS dose (OR, 1.00; 95% CI, 1.00-1.01; *P* = .031). Within the nocturnal cough asthma group, individuals with poorly controlled asthma had higher cough frequency (*P* = .04). We show diurnal variation of cough in asthma, with a nadir in the early hours of the morning when one might expect symptoms to be greatest due to increased airway narrowing^2^ and airway inflammation.^3^ We also show a diurnal variation in cough frequency in healthy individuals, suggesting that the physiological circadian mechanism controlling cough in asthma is intact. The decrease in cough frequency at the expected onset of sleep and the significant increase in cough frequency on expected waking suggest a central suppressive effect of sleep on cough^8^ in asthma or a raised threshold for cough reflex activation during sleep.^9^ The main limitations of this study are the secondary analysis of preexisting data, and that groups were not matched well on age; however, age is not known to significantly influence cough frequency. Nonetheless, these data suggest that further prospective studies are warranted, although only studies in constant conditions and controlling for the effect of sleep would allow one to determine whether the endogenous circadian clock plays a role in the diurnal rhythm of cough frequency.

We identified that patients with asthma with nocturnal cough use more frequent and increased doses of ICS and have poorer asthma control. Despite this, these patients are still coughing, implying that currently used inhaled treatments may not be effective at targeting cough. Commonly used asthma symptom questionnaires place emphasis on night symptoms being a marker of worsening asthma and often lead to escalation in ICS. On the basis of our data, perhaps clinicians should place more emphasis on cough frequency on waking and cough-suppressant drugs might be more efficacious if active on waking.
